# Massive Hemoptysis in a Case of Intralobar Pulmonary Sequestration Associated with Pulmonary Hypoplasia and Meandering Right Pulmonary Vein: Diagnosis and Management

**DOI:** 10.1155/2012/960948

**Published:** 2012-10-24

**Authors:** Manoranjan Mohapatra, Sanjeet Mishra, Paresh Jena

**Affiliations:** ^1^Department of Radiology, Pradyumna Bal Memorial Hospital, Kalinga Institute of Medical Sciences, KIIT Campus-5, Patia, Orissa, Bhubaneswar 751024, India; ^2^Department of Medicine, Kalinga Hospital, Orissa, Bhubaneswar 751023, India

## Abstract

Pulmonary sequestration is a congenital malformation characterized by focal area of dysplastic lung tissue that lacks normal communication with tracheobronchial tree and receives blood supply from systemic arteries. Surgical resection has been the conventional method of treatment of pulmonary sequestration. In recent years transarterial embolization of the anomalous systemic arteries has emerged as a suitable alternative to surgery. In this paper, we describe transarterial coil embolization for control of massive life-threatening hemoptysis in a rare case of intralobar sequestration in right lung associated with ipsilateral pulmonary hypoplasia and meandering right inferior pulmonary vein. A 3-year follow-up computed tomographic (CT) angiography revealed complete regression of the sequestration along with altered pulmonary arterial contour. To the best of our knowledge, transarterial coil embolization for control of massive life-threatening hemoptysis in such a complex pulmonary anomaly has not yet been reported.

## 1. Introduction

Intralobar sequestration is the most common type of pulmonary sequestration. The conventional method of treatment of pulmonary sequestration has been surgical resection. In recent years few cases of involution of pulmonary sequestration following embolization in adults have been reported. We describe complete regression of intralobar sequestration following transcatheter coil embolization of anomalous systemic arteries in an adult who presented with massive hemoptysis. The intralobar sequestration in our case coexisted with ipsilateral pulmonary hypoplasia and meandering right inferior pulmonary vein. There were morphological changes in right pulmonary artery on a follow-up CT study, which has not been described earlier.

## 2. Case Report

A 28-year male, nonsmoker, presented to the emergency department of our hospital with history of massive hemoptysis (500 mL to 600 mL per day), which started 3 days prior to presentation. It was not associated with chest pain, dyspnea, loss of body weight, fever, or swelling of lower limbs. He had no history of bleeding disorder or anticoagulation therapy. He had history of an episode of cough with fever at the age of 2 years, which had subsided with treatment, but the records were not available.

**Figure 1:**
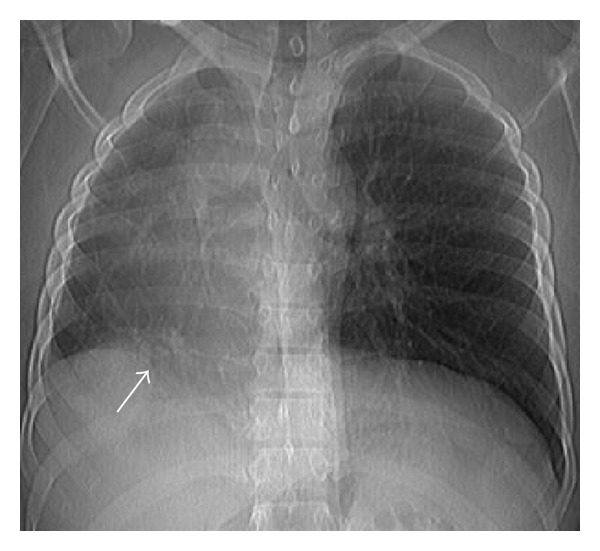
Chest radiograph depicts consolidation at right lower zone (white arrow) in addition to reduced right lung volume and ipsilateral shifting of cardiac shadow.

**Figure 2:**
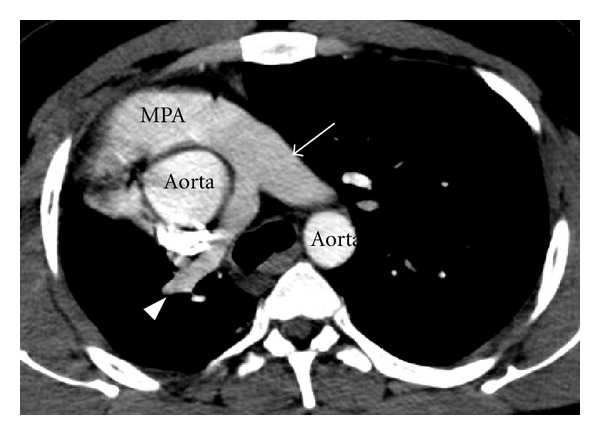
CT angiography axial image depicts smaller calibre right pulmonary artery (arrow head), normal calibre left pulmonary artery (thin arrow), main pulmonary artery (MPA).

**Figure 3:**
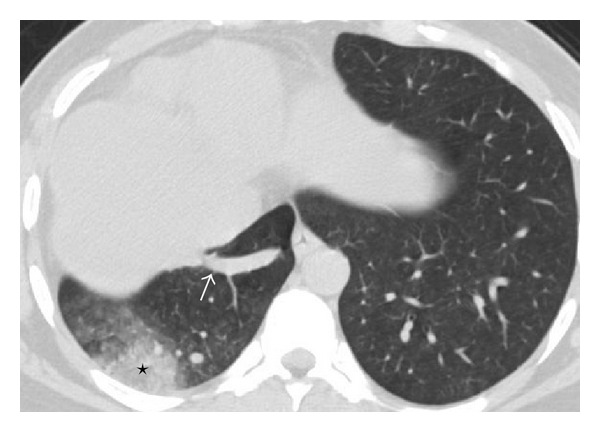
CT axial image depicts sequestration as patchy consolidation at right posterobasal region (asterix), meandering right inferior pulmonary vein (arrow).

**Figure 4:**
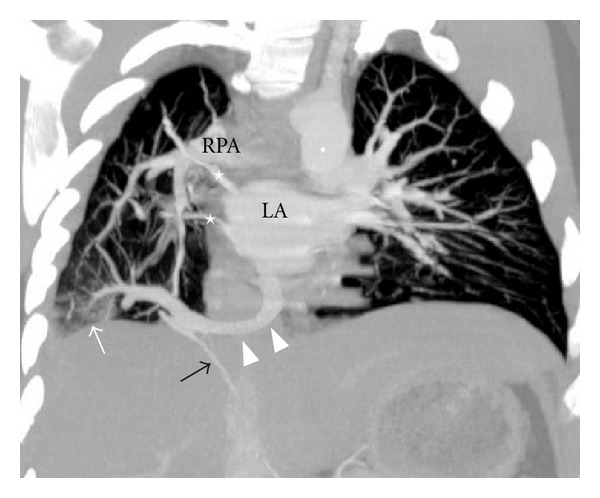
CT angiography maximum intensity projection image in coronal plane depicts right superior and middle pulmonary vein (asterix). Sequestration (white arrow), meandering right inferior pulmonary vein (arrow heads). Anomalous systemic arterial supply (black arrow). Left atrium (LA). Right pulmonary artery (RPA).

**Figure 5:**
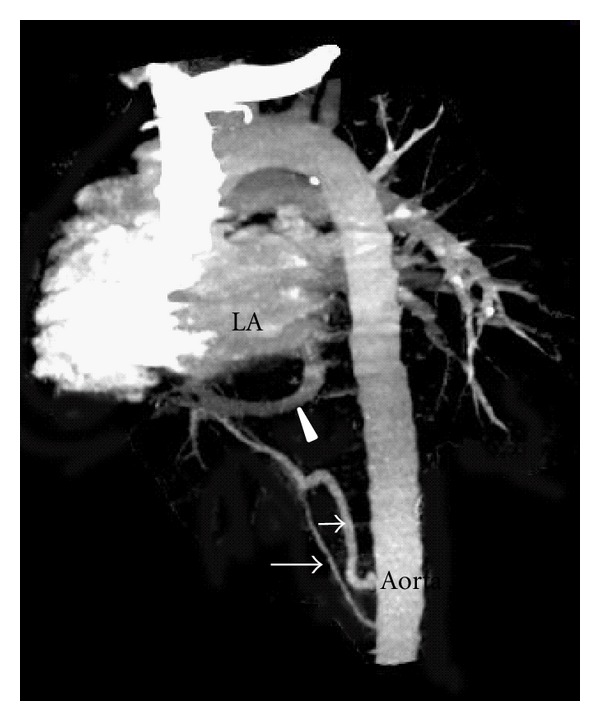
CT angiography volume rendered image depicts two anomalous arterial supply to the sequestration (arrows) and meandering right inferior pulmonary vein (arrow head).

**Figure 6:**
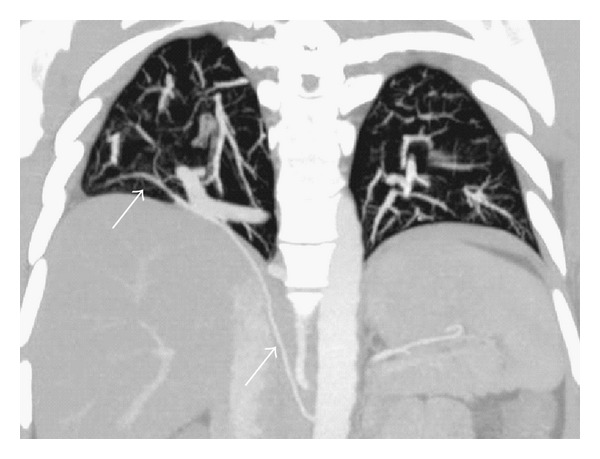
CT angiography maximum intensity projection image in coronal plane depicts anomalous arterial supply (arrow) to the normal lung tissue contiguous with sequestration.

Chest radiograph revealed reduced volume of right lung with patchy consolidation at lower zone and ipsilateral shifting of cardiac shadow (Figure 1). Echocardiography revealed mild left ventricular dysfunction without any congenital cardiac anomaly. Noncontrast CT scan of thorax followed by CT angiography of thorax and upper abdomen was performed for further evaluation of the above abnormality. The CT images revealed absence of right lung upper lobe parenchyma as well as its bronchus, absence of middle lobe parenchyma with blind-ending rudimentary bronchus, and presence of lower lobe parenchyma as well as its bronchus. The right pulmonary artery was patent but smaller in caliber compared to the left one (Figure 2). All the basal segments of right lower lobe, their corresponding bronchi, and accompanying pulmonary arterial branches were present. A patchy consolidation at the posterobasal region of right lung was noted which was supplied by two anomalous systemic arteries (Figures 3, 4, and 5). These two anomalous arteries were arising from abdominal aorta, the larger one just cranial and the smaller one caudal to the celiac axis origin. A branch from one of the above anomalous systemic arteries was also supplying the contiguous normal lung parenchyma at anterior and lateral basal segments (Figure 6). There was no pulmonary arterial branch supplying the sequestration. The venous drainage from upper and mid part of right lower lobe was via small right upper and middle pulmonary veins which joined the right inferior pulmonary vein just before its entry into left atrium. The venous drainage from the sequestration as well as the right lower lobe was exclusively into left atrium via dilated right inferior pulmonary vein, which had a circuitous course through lung parenchyma towards right cardiophrenic angle and emptied into left atrium after a U-turn (Figures 4 and 5). Neither the lower lobe nor the pneumonic patch had venous drainage into IVC, right atrium, hepatic vein, or portal vein.

 There was dextroversion and shifting of mediastinal structure to the right side. The left lung was structurally normal but had compensatory hyperinflation. The left pulmonary artery was normal in caliber. There was no musculoskeletal, diaphragmatic, renal, cardiac, or gastrointestinal tract anomaly. Based on the above findings the diagnosis of intralobar sequestration with pulmonary hypoplasia having meandering right inferior pulmonary venous drainage into left atrium was made.

**Figure 7:**
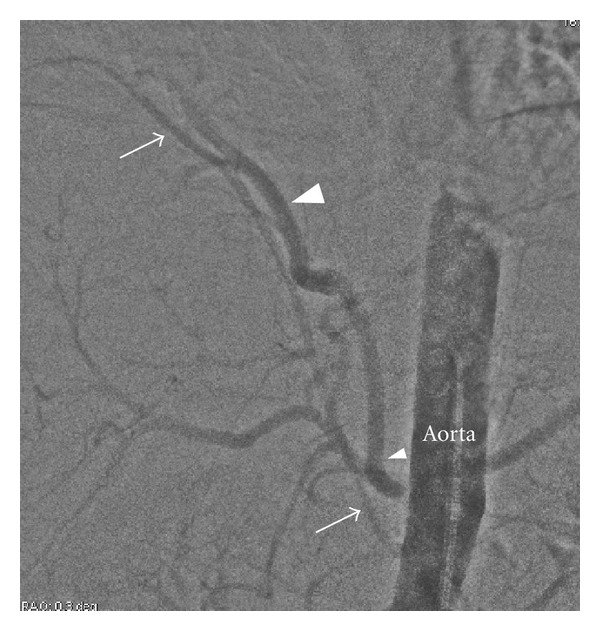
Intra-arterial digital subtraction angiogram reveals two anomalous arteries originating from abdominal aorta (arrow and arrow head).

**Figure 8:**
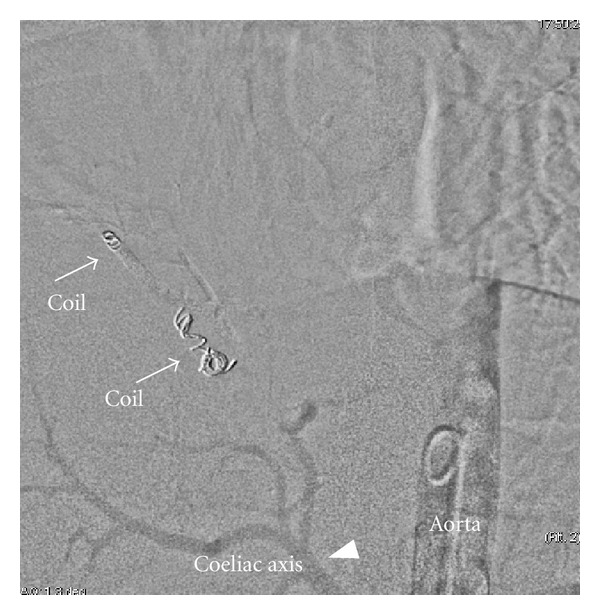
Intra-arterial digital subtraction angiogram following coil deployment depicts complete occlusion of anomalous arteries (arrows). Normal coeliac axis shown (arrow head).

**Figure 9:**
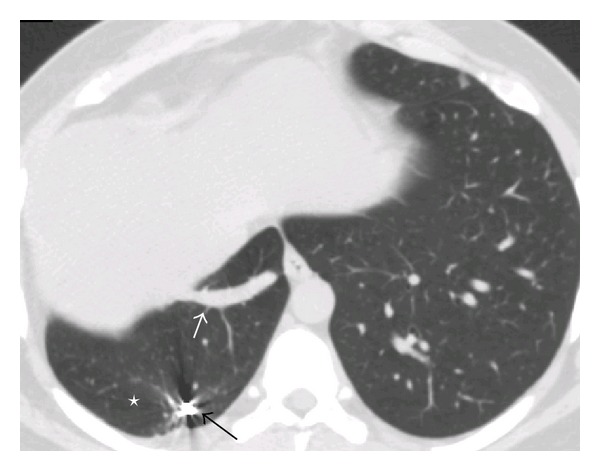
CT axial image depicts complete regression of the sequestration at right posterobasal region (asterix), meandering right inferior pulmonary vein (white arrow). Artefact from the coil within one of the anomalous systemic artery (black arrow).

**Figure 10:**
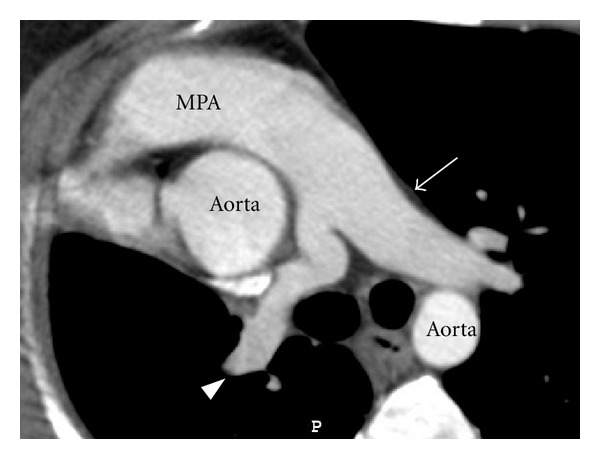
CT angiography axial image depicts contour abnormality of right pulmonary artery (arrow head), Normal caliber left pulmonary artery (thin arrow), Main pulmonary artery (MPA).

He was taken up for emergency transcatheter coil embolization of the anomalous arteries feeding the sequestration. Catheter angiography revealed two anomalous arteries from abdominal aorta feeding the sequestration (Figure 7). The diameters of the larger and the smaller caliber anomalous arteries were 5 mm and 2.8 mm, respectively. The venous drainage from the sequestration was exclusively into left atrium via meandering right inferior pulmonary vein. There was no arteriovenous fistula. Two nester coils (cook) of 6 mm diameter and 14 cm length in the larger and one 4/2 tornado coil (cook) in the smaller caliber anomalous arteries were deployed. Check angiogram (after 15 minutes of coil deployment) revealed complete occlusion of both these arteries (Figure 8). The hemoptysis resolved subsequently. He was discharged 2 days later. The patient was asymptomatic following embolization. A 3-year follow-up CT angiography revealed complete regression of the sequestration (Figure 9). However, the right pulmonary artery showed contour abnormality and kinking (Figure 10). Pulmonary artery catheterization was suggested for further evaluation, but the patient did not comply.

## 3. Discussion

Pulmonary sequestration which constitutes 0.15–6.4% of all congenital pulmonary malformation is of two types: intralobar and extralobar [1]. The incidence of intralobar sequestration is 83.95% of all pulmonary sequestrations [2]. Intralobar pulmonary sequestration consists of nonfunctioning lung tissue that lacks normal communication with the tracheobronchial tree, receives systemic arterial supply, and shares common visceral pleura with the parent lobe. The extralobar sequestration has its own pleural covering. Venous drainage is different in the two types of pulmonary sequestration making it possible to differentiate them [3, 4]. In an intralobar sequestration the anomalous venous drainage is typically via the pulmonary vein, whereas in an extralobar sequestration the anomalous venous drainage is into systemic vein, commonly the azygous vein, although unusual drainage into portal vein or subclavian vein has been reported [3]. Radiographically the dysplastic tissue can manifest as a mass lesion, cystic lesion, cavitary lesion, pneumonic lesion, or bronchiectasis and very rarely it may appear as intrapulmonary cord like shadow or atelectasis [2]. In our case there was pneumonic type of lesion in the posterobasal region of right lower lobe with two anomalous arterial feeders originating from upper abdominal aorta. The venous drainage in this case was through right inferior pulmonary vein having abnormal circuitous intraparenchymal course before opening into left atrium suggestive of meandering pulmonary vein [5]. Based on these findings the diagnosis of intralobar sequestration with pulmonary hypoplasia and meandering right inferior pulmonary vein was made. The possibility of Scimitar syndrome was ruled out in this case as there was no anomalous pulmonary venous drainage from right lung into inferior vena cava. Since the patient was scheduled for arterial embolization following the radiological diagnosis, bronchoscopy was not attempted, thereby the risks of possible airway compromise from sedation and delay in definitive treatment associated with bronchoscopy were avoided [6].

Patients with pulmonary sequestration may be asymptomatic or can present with chest pain, dyspnea, recurrent chest infection or fatal massive hemoptysis [7]. Although the aetiology of the hemoptysis is uncertain, it is thought to be due to high-pressure blood flow in the sequestered lung from the anomalous systemic arteries [8, 9]. 

Identification of anomalous arterial supply to a sequestration is essential prior to treatment either by surgery or by embolization procedure. The commonest site of origin of the anomalous arteries is from thoracic aorta (76.55%) followed by abdominal aorta (18.47%) [2]. Though the incidence of multiple arterial supply (20.91%) is less common than single arterial supply (79.09%), it is essential to identify all of them so as to prevent massive intraoperative hemorrhage due to transection of unidentified vessel [2, 3]. Similarly failure to occlude all anomalous arterial feeders during transcatheter arterial embolization procedure may lead to incomplete devascularisation of the sequestration and recurrence of hemoptysis. In the present case 3-dimensional volume rendered reconstructed images from CT angiography clearly demonstrated the origin, course, and caliber of each of the two anomalous systemic arterial feeders from abdominal aorta.

 The conventional method of treatment of pulmonary sequestration has been surgical resection, which is associated with morbidity and complications. Embolization has been previously reported as a safe alternative to surgery in pediatric patients [10]. Few reports on embolization of pulmonary sequestration in adults have been described in English literature [9, 11]. The present case had right lung agenesis-hypoplasia complex, where only the right lower lobe lung parenchyma was developed but it was harbouring the intralobar sequestration. Massive hemoptysis in this case prompted us for a less invasive procedure like transcatheter coil embolisation of the feeding arteries. The option of lobectomy of the right lower lobe in this case was reserved for recurrence of hemoptysis or development of infection in the sequestration. However no such complications have been encountered and there was complete resolution of the sequestration on a 3-year follow-up CT angiography study. 

It is thought that embolization causes thrombosis of the feeding artery, which results in reduced perfusion, loss of vascularity, and progressive infarction of the anomalous lung tissue which finally becomes fibrosed. This leads to reduction in the size of the sequestration and sometimes its involution [9]. Complete regression of pulmonary sequestration following embolization in adults has been reported in two cases; however none of them had such complex pulmonary anomaly [11, 12]. 

The right pulmonary artery revealed contour abnormality and kinking on follow-up CT angiography study which were not seen on baseline study. We presume that hemodynamic changes in the pulmonary circulation following the occlusion of anomalous systemic arteries could have led to flow alteration in the right pulmonary artery causing the morphological changes as described previously. Few reports on reduction in pulmonary arterial pressure following surgery for intralobar sequestration and sequestration spectrum (anomalous systemic arterial supply to normal basal lung segment) have been mentioned in the literature [13–15]. However hemodynamic alteration in pulmonary artery following transarterial embolization needs to be confirmed by further studies.

In conclusion, the present case describes a rare combination of intralobar sequestration with meandering right inferior pulmonary vein and pulmonary hypoplasia where transarterial embolization was performed for massive life threatening hemoptysis, resulting in complete resolution of the sequestration. To the best of our knowledge, till date no such case has been reported in the English literature.
